# The Best Inheritance

**Published:** 1857-12

**Authors:** 


### THE BEST INHERITANCE

Is ability to help one's self, manly principles, and a good constitution. Infinitely more valuable are these than beauty, birth, or blood. Beside them, wealth, and fame, and position, pale away in darkness, when they have come down from father to son; because then, they may be lost, and are ignobly lost in countless instances. But with these—health, manliness, and self-sustaining power—wealth is created, a name may be founded as lasting as that of the Cæsars, and a standing among men secured of more honorable mention than the coffers of all kings could purchase.

These things being true, the wiser policy of parents is, not to work themselves to death, in order to leave their children perishable thousands; but, by judicious teachings from infancy, show those children how to take care of their health, and how to make a living for themselves.

